# Antioxidant action and potential neuroprotection of polyphenolics extracted from *Astragalus membranaceus* residue

**DOI:** 10.3389/fnut.2025.1621848

**Published:** 2025-07-23

**Authors:** Lu Li, Qiaona Wang, Ying Cao, Jianmei Li, Yulong Wu, Chun Hua, Feng Zhou, Shengjie Li, Su Liu

**Affiliations:** ^1^School of Food Science and Pharmaceutical Engineering, Nanjing Normal University, Nanjing, China; ^2^School of Food Science, Nanjing Xiaozhuang University, Nanjing, China; ^3^School of Ecology and Applied Meteorology, Nanjing University of Information Science and Technology, Nanjing, China

**Keywords:** *Astragalus membranaceus* residue, neuroprotection, phenolic compounds, oxidative stress, antioxidant

## Abstract

**Introduction:**

Polyphenols, recognized as nutritional supplements, have emerged as promising therapeutic agents for various diseases, particularly brain disorders. However, due to the limitation of the extraction method, *Astragalus membranaceus* residues (AR) retain substantial bound phenolics with unexplored neuronal antioxidant activity.

**Methods:**

In this study, free, esterified, and bound phenolic compounds were sequentially extracted from AR. Specific compounds in the three phenolic fractions were identified using ultra-high-performance liquid chromatography coupled with quadrupole time-of-flight mass spectrometry and categorized into phenolic acids, flavonoids, and isoflavonoids. Antioxidant efficacy was comparatively evaluated through free radical-scavenging assays, ferric reducing antioxidant power assays, and *in vitro* neuroprotective assessments using PC12 cell models.

**Results:**

The insoluble-bound fraction had the highest total phenolic content, followed by free and esterified phenolics. Bound phenolic compounds contained the highest amounts of flavonoids. The bound phenolic fraction demonstrated superior comprehensive antioxidant capacity. An *in vitro* neuroprotective assessment using H_2_O_2_-stimulated PC12 neuronal cells demonstrated that the bound phenolic fractions significantly relieved oxidative stress, as evidenced by an increase in superoxide dismutase and catalase and a reduction in intracellular reactive oxygen species and malondialdehyde compared to untreated controls. Bound phenolics in AR also reduced the expression of oxidative stress-related genes in PC12 cells.

**Discussion:**

This study suggests that BP in AR may benefit neurological and brain health as potential nutritional therapies.

## 1 Introduction

For *Astragalus membranaceus,* a pivotal medicinal and culinary plant, exhibits a plethora of biological activities, including immunomodulatory, antioxidant, and antihypertensive effects. It possesses remarkable therapeutic efficacy in brain health, hepatic injury repair, tumor suppression, and glycemic regulation (1, 2). The antioxidant capacity of the plant is primarily attributed to its phenolic constituents. Polyphenols can be used as nutritional supplements and are a potential therapeutic option for various diseases (3, 4). A study on the role of nutrition in Parkinson’s disease (PD) found that foods rich in polyphenols were able to reduce the occurrence of PD and slow clinical progression (5). Emerging evidence indicates that polyphenols, a specific nutrient, have a beneficial effect on brain health (6). Characteristic polyphenols, including catechin (7–9), sanguinarine (10), and hesperidin (11, 12), have been identified as critical contributors to the overall antioxidant effects because of their superior free radical-scavenging capabilities and redox-modulating activities (13).

Phenolic compounds in food matrices exist predominantly in three forms: free phenolics (FP), esterified phenolics (EP), and insoluble-bound phenolics (BP). When subjected to the principal preparation method for *A. membranaceus* for medicinal formulations and culinary applications (traditional water decoction), water-soluble free phenolics became the primary bioaccessible fraction. However, a significant proportion of EP and insoluble BP are retained within the discarded *A. membranaceus* residue (AR) through covalent interactions, including ester, glycosidic, and ether bonds, with macromolecules, such as cellulose, proteins, polysaccharides, lipids, and components of plant cell walls (14). This structural complexity restricts their extraction efficiency and bioavailability, which are critical challenges for the comprehensive use of phytochemicals (15).

Oxidative stress is a pathological process where the body experiences harmful stimuli, leading to an imbalance between oxidation and antioxidant systems. This results in excessive production of reactive oxygen species (ROS) and reactive nitrogen species (RNs), which surpass the capacity of intracellular antioxidants and cause cell and tissue damage (16). Under normal conditions, ROS production and antioxidant defenses are in dynamic balance, maintaining intracellular redox homeostasis, which is crucial for normal cell metabolism, signaling, and other functions. However, exposure to pathological factors such as alloxan, heavy metals, inflammatory agents, radiation, and neurodegenerative diseases can disrupt this balance. Consequently, ROS accumulates excessively, initiating oxidative stress reactions.

In the nervous system, oxidative stress is closely related to neurotoxicity and is a key factor leading to nerve cell injury, neurological dysfunction, and the occurrence and development of neurodegenerative diseases (17). Nerve cells are rich in polyunsaturated fatty acids and are highly sensitive to oxidative damage. When oxidative stress occurs, excessive ROS will attack the lipids on the neuronal cell membrane, trigger lipid peroxidation, damage the integrity and fluidity of the cell membrane, affect the uptake and release of neurotransmitters and the function of ion channels, and then interfere with nerve signaling, resulting in neurotoxic effects (18).

Studies have shown that food byproducts can be used as rich biological resources to create economic value. For example, wheat bran can reduce chronic diseases such as obesity and diabetes (19), fruit and vegetable byproducts can be used as animal feed (20), and the phenolic compounds in olive leaves can be used as food additives (21). Phenolic compounds are widely used antioxidants.

Recent studies have highlighted the superior antioxidant efficacy of bound phenolics (BP) over free and esterified phenolics across diverse biological systems. Cereal grains, such as wheat and maize, contain BP that exhibit radical-scavenging activity and inhibit lipid peroxidation (22). Notably, berry species (blackberries, black raspberries, blueberries) demonstrate potent inhibition of copper-induced LDL oxidation and radical-mediated DNA damage, an effect attributed to their BP acid fractions—activity exceeding that of FP and EP (23). *In vitro* studies show chickpea-derived BP provides enhanced protection against H_2_O_2-_induced oxidative damage in HuH-7 hepatocytes compared to other phenolic forms (24), while cocoa bean hulls rich in BP exhibit significant antioxidant capacity, positioning them as viable nutraceutical ingredients (25). Mechanistically, BP from rice bran and adzuki beans modulate glycemic responses via gut microbiota regulation and insulin signaling activation in skeletal muscle (26). The collective findings demonstrate that the structural stability and controlled release kinetics of BP, in contrast to the rapid bioavailability of their free forms, enable their dual application as sustained-release antioxidants in food systems and modulators of intestinal homeostasis in the pharmaceutical context. Consequently, the strategic valorization of insoluble-bound phenolics in AR represents a scientifically grounded approach for enhancing resource utilization efficiency and creating value-added bioactive products.

In this study, free phenolics, esterified phenolics, and insoluble-bound phenolics from AR, an *A. membranaceus* byproduct, were extracted using various organic solvents. Ultra-high-performance liquid chromatography-quadrupole time-of-flight mass spectrometry (UHPLC Q-TOF-MS) was used to identify phenolic species. Subsequently, the *in vitro* antioxidant capacity of the three polyphenolic fractions was systematically evaluated, with primary assessment parameters including ABTS and DPPH radical-scavenging activities as well as ferric ion reducing power. An *in vitro* neuroprotective assessment using H_2_O_2_-stimulated PC12 neuronal cells demonstrated that the bound phenolic fractions significantly relieved oxidative stress, as evidenced by an increase in superoxide dismutase (SOD) and catalase (CAT) and a reduction in intracellular ROS and malondialdehyde (MDA) compared to untreated controls. Also, BP in AR can downregulate the expression of intracellular genes related to oxidative stress. The systematic development of BP in AR holds the potential to significantly improve overall resource utilization efficiency and concurrently contribute to the theoretical foundations and technological frameworks essential for developing natural nutritional supplements.

## 2 Materials and methods

### 2.1 Materials and reagents

*Astragalus membranaceus* was provided by Jiangyin Tianjiang Pharmaceutical Company. 2,2′-azinobis(3-ethylbenzothiazoliline-6-sulfonic acid), diammonium salt (ABTS), 1,1-diphenyl-2-bitterhydrazine (DPPH), and plant total phenolic content (TPC) detection kits (micro method) were purchased from Beijing Solaibao Technology Co., Ltd. DMEM medium was purchased from Thermo Fisher Scientific Technologies. Fetal bovine serum was purchased from Nanjing Shenghang Biotechnology Co., Ltd. 0.25% Trypsin–EDTA was purchased from Thermo Fisher Technology LLC. The penictomycin mixture was purchased from Beijing Solaibao Technology Co., Ltd. PBS buffer solution was purchased from Jiangsu KGI Biotechnology Co., Ltd. CCK-8 kits were purchased from Shanghai Biyuntian Biotechnology Co., Ltd.

Ultra-high performance liquid chromatography was produced by Waters (United States), 6,600 QTOF was produced by AB SCIEX, and ACQUITY UPLC HSS T3 (1.8 μm, 2.1 × 100 mm) was produced by Waters. The solvents, including HPLC acetonitrile and methanol, were purchased from Merck (Darmstadt, Germany). The formic acid was purchased from CNW (Shanghai, China).

### 2.2 Instruments and equipment

PX224ZH electronic analytical balance was purchased from OHAUS Instruments Ltd.; the DFY-5000 swing high-speed crusher was purchased from Wenling Linda Machinery Co., Ltd.; the JJ1000Y electronic balance was purchased from Changshu Shuangjie Testing Instrument Factory. SB-5200DTD ultrasonic cleaner was purchased from Ningbo Xinzhi Biotechnology Co., Ltd. RE-2000B rotary evaporator was purchased from Shanghai Yarong Biochemical Instrument Factory. HWS-24 Electric Constant Temperature Water Bath was purchased from Shanghai Huitai Instrument Manufacturing Co., Ltd.; the RE-3000 rotary evaporator and SHZ-III. Circulating multi-purpose vacuum pumps were purchased from Shanghai Yarong Biochemical Instrument Factory.

### 2.3 Raw material pretreatment

The AR was crushed into powder with a high-speed pulverizer.

### 2.4 Extraction and determination of free, esterified, and bound phenolics

Free, esterified, and bound phenolics were extracted from AR following the method outlined by Dos Reis et al. (27) with slight modification. Briefly, the AR (20 g) was extracted with 300 mL of 50% ethanol for ultrasound at room temperature (25°C) for 30 min in a water bath. Then, the solution was concentrated on a rotary evaporator at 40°C to remove the organic solvent. Then, the pH of the concentrated solution was adjusted to 2 with 6 M hydrochloric acid (HCl), and the filtrate was extracted with n-hexane (1:1, v/v). The remaining aqueous phase was extracted with ethyl acetate and anhydrous ether (1:1, v/v). The anhydrous sodium sulfate was added until it did not fall in lumps, and the filtrate was concentrated on a rotary evaporator at 40°C. Finally, free phenolics were dissolved in 3 mL of methanol to obtain free phenolics.

The bound phenolics in the filter residue were hydrolyzed with NaOH (4 M) for 4 h in the dark. The pH of the solution was adjusted to 2 with 6 M HCl, and the filtrate was extracted with n-hexane (1:1, v/v), and the bound phenolics were extracted with ethyl acetate and anhydrous ether (1:1, v/v) in the same way as explained above.

The esterified phenolics in the aqueous solution were hydrolyzed with 4 M NaOH, and 6 M HCl was added to adjust the pH to 2. Then, the filtrate was extracted with n-hexane (1:1, v/v), and the esterified phenolics were extracted with ethyl acetate and anhydrous ether (1:1, v/v) in the same way as explained above.

### 2.5 Determination of total phenolic content

Total phenolic content (TPC) was measured according to the instructions of the kit purchased. Firstly, the gallic acid standard solution was diluted into 7 concentration gradients with distilled water. Reagents were sequentially introduced into the assay tube in the specified order, and the absorbance was measured at a wavelength of 760 nm.

### 2.6 UHPLC Q-TOF-MS

The method of UHPLC Q-TOF-MS is mainly based on Guo et al. and Bianchi et al. (4, 5) and has been slightly modified. The UHPLC Q-TOF-MS system consists of an ultra-high performance liquid chromatography (Waters, United States) with ACQUITY UPLC HSS T3 (1.8 μm, 2.1 × 100 mm), hyphenated to a quadrupole-time of flight equipped with electrospray ionization (ESI). The ESI analysis was performed both in positive (5,500 V) and negative (−4,500 V) polarity. The mobile phase consisted of A (0.1% formic acid in water) and B (0.1% acetonitrile format solution). The gradient conditions were as follows: 0–1.50 min/95% A, 2.50–14.00 min/90–60% A, 22.00 min/5% A, 26.0–30.00 min/95% B. The mass spectrometer parameters included a capillary voltage of 30 eV, a column temperature of 40°C, and a sample load is 4 μL.

The relative content of phenolic compounds is determined using peak area normalization, whereby the peak area of the target compound is expressed as a percentage of the total peak area. Relative content (%) = (Ai/ΣAi) * 100%, Ai, peak area of the target compound; ΣAi, total peak area of all detected compounds.

### 2.7 Antioxidant activity

#### 2.7.1 2,2-diphenyl-1-picrylhydrazyl radical scavenging activity

2,2-diphenyl-1-picrylhydrazyl (DPPH) radical scavenging activity of free, esterified, and bound phenolics was measured by a reported method (28) with slight modification. L-Ascorbic acid (Vc) was used as a positive control. 100 μL of different concentration gradients of phenolics and Vc were mixed with DPPH or anhydrous ethanol in a 96-well plate. At the same time, a mixture of anhydrous ethanol and DPPH was the blank control. Absorbance at 517 nm was measured spectrophotometrically after 30 min.

#### 2.7.2 2,2′-azino-bis(3-ethylbenzothiazoline-6-sulfonic acid) radical scavenging activity

2,2′-azino-bis(3-ethylbenzothiazoline-6-sulfonic acid) (ABTS) radical scavenging activity of free, esterified, and bound phenolics was measured by a reported method (29) with slight modification. Vc and samples were diluted into the same concentration gradients as explained above. 160 μL of different concentration gradients of phenolics and Vc were mixed with ABTS working fluid (40 μL) in a 96-well plate. At the same time, a mixture of purified water and ABTS working fluid was the blank control. Absorbance at 734 nm was measured spectrophotometrically.

#### 2.7.3 Ferric reducing/antioxidant power assay

Briefly, the Ferric reducing/antioxidant power (FRAP) assay contained 0.2 mol/L phosphate buffer (pH 6.6), 10 g/L K_3_[Fe(CN)_6_], 100 g/L trichloroacetic acid (TCA), and 1 g/L FeCl_3_. Ferric reducing/antioxidant power (FRAP) assay was performed according to a reported method (29). Samples and Vc solution were placed in 5 mL centrifuge tubes, phosphate buffer and K_3_[Fe(CN)_6_] were sequentially added to the tubes, after a water bath at 50°C for 30 min, TCA was added to the solution. Then, the supernatant was taken and mixed with pure water and FeCl_3_ for 15 min in the dark. Finally, absorbance at 734 nm was measured spectrophotometrically. The reducing power was determined to be proportional to the absorbance, based on the established relationship.

### 2.8 Cytotoxicity and activity assays

PC12 cells were cultured in DMEM containing 10% FBS (fetal bovine serum) and 0.1% penicillin–streptomycin solution and grown at 37°C in 5% CO_2_ incubator. The cell culture medium was changed every 2 days. Cellular toxicity and activity after the sample were determined by the CCK-8 assay (30). Briefly, cells were inoculated into 96-well plates at a density of 3 × 10^5^ cells/mL for 24 h. The cell culture medium was then removed, and PC12 cells were incubated for 24 h using five different concentrations of phenolics (1–10,000 μg/mL). After the removal of the medium, 10% CCK-8 solution was added to each well. Following a 20-min incubation, absorbance at 425 nm was measured using a BioTek Synergy HTX multi-mode microplate reader.

#### 2.8.1 Intracellular reactive oxygen species levels

The method of measuring reactive oxygen species (ROS) levels has been modified according to Dominiak et al. (31). PC12 cells were incubated in 96-well plates at 3 × 10^5^ cells/well for 24 h. 100 μL of the three phenolics (100 μg/mL) was then added to the wells and incubated for 24 h. The medium was discarded, and the cells were treated with or without 1 mM H_2_O_2_. After 3 h of incubation, the medium was discarded, and diluted fluorescent dye was added to the wells. Intracellular ROS levels were detected by fluorescence microscopy using an Olympus CKX53 inverted microscope.

#### 2.8.2 Intracellular malondialdehyde production

The measurement of malondialdehyde (MDA) levels was modified following a previous protocol (32). PC12 cells were incubated in 6-well plates at 3 × 10^5^ cells/well for 24 h. Samples (100 μg/mL) were then added to the wells and incubated for 24 h. The medium was discarded, and the cells were treated with or without 1 mM H_2_O_2_. Following a 3-h incubation period, the cells were harvested. The measurement of MDA was performed per the instructions of the purchased kit. Absorbance measurements at 532 nm and 600 nm were performed using a BioTek Synergy HTX multi-mode microplate reader.

#### 2.8.3 Intracellular superoxide dismutase production

The superoxide dismutase (SOD) activity assay is based on a colorimetric reaction of WST-8 to detect superoxide dismutase activity in cells (33). The samples were treated the same method as performed above and the measurement of SOD production was carried out according to the instructions of the purchased kit. Absorbance at 560 nm was measured using a BioTek Synergy HTX multi-mode microplate reader.

#### 2.8.4 Intracellular catalase production

Catalase (CAT) activity is measured by reacting with hydrogen peroxide using a colorimetric assay (34). The samples were treated in the same way as described above, and the measurement of CAT production was performed per the instructions of the purchased kit. Absorbance at 520 nm was measured using a BioTek Synergy HTX multi-mode microplate reader.

### 2.9 qRT-PCR to analyze mRNA expression

After PC12 cells were lysed by trizol (Vazyme, Nanjing, China), RNA was extracted from them and then reverse transcribed to cDNA per the kit (Vazyme, Nanjing, China), and the gene level was detected by ABI Step One Plus Real-Time PCR System using SYBR Green qPCR kit (Vazyme, Nanjing, China) for RT-PCR. Primers for Mmp9, Mmp2, Sestrin2 (Sesn2), Hao1, Solute Carrier Family 7 Member 11 (Slc7a11), Ras homolog family member H (Rhoh), and Dual Specific Phosphatase 3 (Dusp3) were synthesized by Sangon Biotech (Shanghai, China). Table 1 lists the sequences. Their expression levels were calculated by the 2^−ΔΔCT^ approach with 18S rRNA as the reference.

**Table 1:** Sequences of primers utilized in qRT-PCR.

Gene	Forward (5^′^-3′)	Reverse (5′-3′)
Mmp9	CAAACCCTGCGTATTTCCATTCATC	GATAACCATCCGAGCGACCTTTAG
Mmp2	ACCAAGAACTTCCGACTATCCAATG	GTACCAGTGTCAGTATCAGCATCAG
Sesn2	CTATATCAAGACGGTTGCCTGCTAC	TCTCTGAGTGGCGGAAGTGC
Hao1	ACCTCACTGCCCATTGTTGTAAAG	TAAGATCCCATCCACACCATGTTTAAC
Slc7a11	CATCATCATCGGCACCGTCATC	TCCACAGGCAGACCAGAACAC
Rhoh	AGGCAGATGTGGTACTAATGTGTTAC	TCCTGACCTCACTAATCCATTTGTTC
Dusp3	GCCAATGACACGCAGGAGTTC	CCTCACGGCAATGGACAAGC
Actin	CTGAGAGGGAAATCGTGCGTGAC	AGGAAGAGGATGCGGCAGTGG

### 2.10 Statistical analysis

Data were analyzed through the GraphPad and Origin 2024. The statistical differences were assessed by analysis of variance (ANOVA) and the differences were considered significant at *p* < 0.05 after applying a t-test. MSDIAL (ver 4.6) software was used to process the data of peak search and peak alignment of the converted abf files, and the databases of Metlin, MassBank, MoNA and HMDB were independently integrated based on the primary and secondary spectrum search, version (V 6.0), and the identification results were obtained.

## 3 Results

### 3.1 FP, EP, and BP content in different extraction methods

As shown in Table 2, the total contents of free, esterified, and bound phenolics were 55, 28, and 105 μg/g (dw), respectively, when ethyl acetate was used as the extraction agent. However, upon mixing ethyl acetate and anhydrous ether (1:1, v/v) as extraction agents, FP’s TPC increased two-fold, EP increased by 1.75 times, and BP increased by 1.4 times. An ethyl acetate/anhydrous ether mixture (1:1, v/v) was selected for use as an enhanced extraction agent in future studies.

**Table 2:** Phenolic content obtained from different extraction solvents.

Extraction solvents	Free	Esterified	Bound
Ethyl acetate (TPC in μg/g dw)	55	28	105
Ethyl acetate and anhydrous ether (TPC in μg/g dw)	128.7	49	149.7

### 3.2 Determination of qualitative and relative content of phenolic compounds using UHPLC Q-TOF-MS

**Figure 1:**
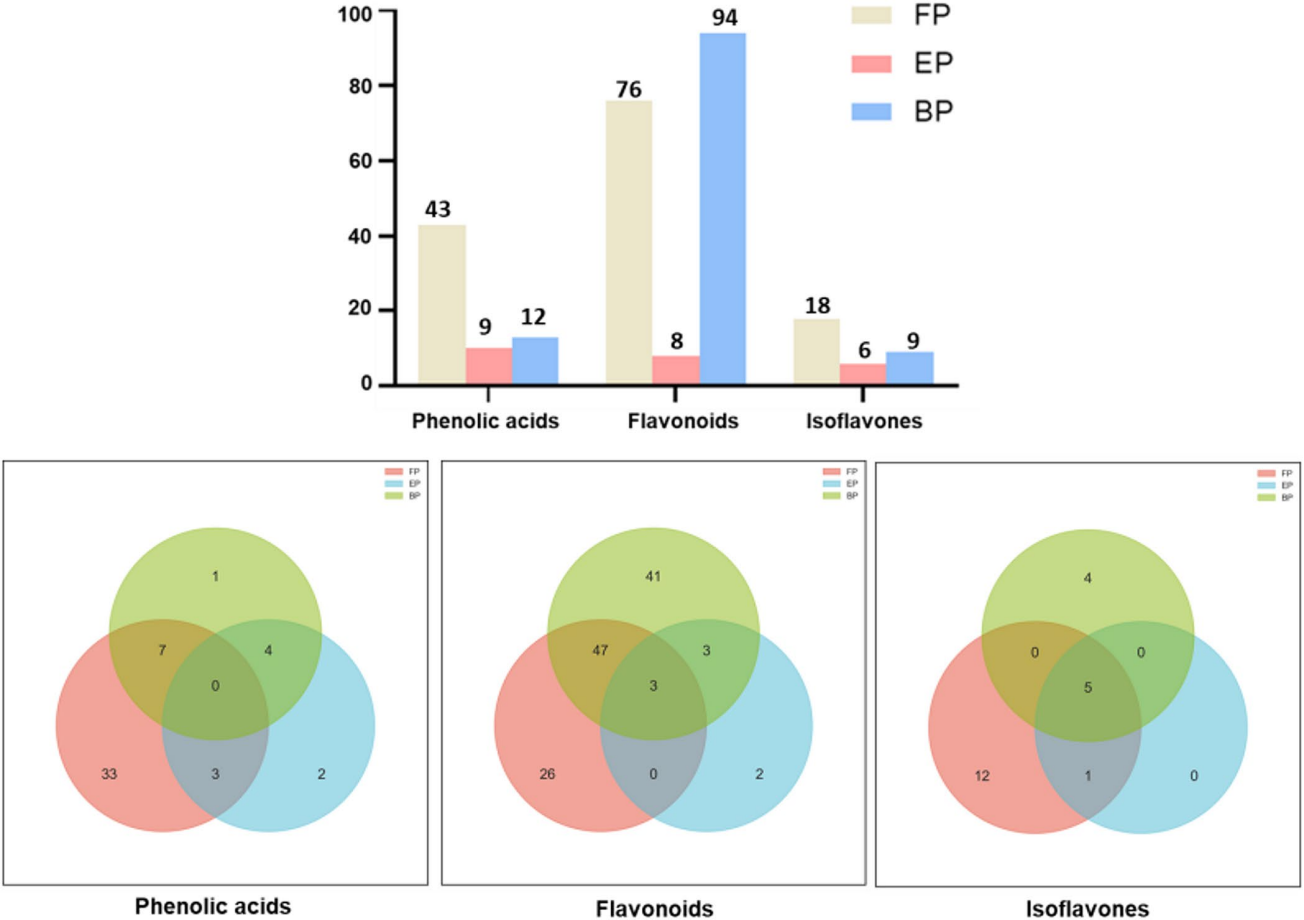
Phenolic composition of different AR extracts. These compounds are classified into three major categories: phenolic acids, flavonoids, and isoflavonoids.

The AR extracts demonstrated great diversity in the phenolic compounds present. UHPLC Q-TOF-MS was used to determine the phenolic compounds in FP, EP, and BP; the qualitative results are shown in Supplementary Table S1. Total ion current (TIC) diagram of the free, esterified and bound phenolics in AR are shown in Supplementary Figures S1, S2. The retention times of the identified phenolics were concentrated at 0–27 min. Supplementary Table S1 shows that FP contained 137 phenolic compounds, EP contained 23 phenolic compounds, and BP contained 115 phenolic compounds. These compounds were classified into three major categories: phenolic acids (FP: 43, EP: 9, BP: 12), flavonoids (FP: 76, EP: 8, BP: 94), and isoflavonoids (FP: 18, EP: 6, BP: 9) (Figure 1). EP was significantly less abundant than FP and BP were. UHPLC Q-TOF-MS revealed a marked divergence in the chemical compositions of FP and BP, wherein FP demonstrated significantly elevated phenolic acids (36 versus 5 species) and isoflavonoids (13 vs. 4 unique compounds), whereas BP manifested greater flavonoid diversity (44 vs. 26 unique compounds) (Figure 1). Evidence shows that the composition of the compounds in the different phenolic fractions varied greatly. Both FP and BP contained 63 phenolic compounds, including phloretin-2’-O-glucoside, epicatechin, isopongaflavone, calycosin, and glyasperin C. Moreover, the relative content of 38 compounds such as Phloretin-2′-O-glucoside, 4′-Hydroxy-5, 7-dimethoxyflavanone, Isosinensetin, Isopongaflavone, etc. in BP was higher than FP. The relative content of phloretin-2′-O-glucoside in BP was 23.27%, whereas that in FP was only 0.47%. Similarly, the relative isopongaflavone content of BP was 28.88%, whereas that of FP was 0.04% (Table 3).

**Table 3:** Relative contents of compounds shared by FP and BP.

Phenolic compounds	FP		BP	
	Area	Relative content (%)	Area	Relative content (%)
Ozagrel	65424.8477	0.06%	136152.156	0.37%
Methyl gallate	1075942.88	1.06%	1043255.56	2.86%
Syringic acid	323878.188	0.32%	115010.828	0.32%
2-Methoxy-4-pentadecylbenzoic acid	1236209	1.22%	3388572	9.29%
Benzamide	101177.977	0.10%	46450.2344	0.13%
Vanillic acid	103968.555	0.10%	41457.082	0.11%
(-)-Epigallocatechin	47748.1445	0.05%	14032.4619	0.04%
Isokaempferide	283983.219	0.28%	45727.1992	0.13%
Astragalin	150041.859	0.15%	11114.918	0.03%
Eriodictyol-7-O-glucoside	10492.042	0.01%	1057443.75	2.90%
7,3′,4′-Trihydroxyflavone	2304591.5	2.28%	127997.82	0.35%
Apigenin-6-C-glucoside-7-O-glucoside	171235.703	0.17%	50054.7305	0.14%
Apigetrin	261777.531	0.26%	9951.3125	0.27%
2′,6′-Dihydroxy-4-methoxychalcone-4’-O-neohesperid	699246.438	0.69%	1753955.63	4.81%
Luteolin 3′,4′-dimethyl ether	833160.875	0.82%	33094.0234	0.09%
Flavanomarein	65434.418	0.06%	64302.3281	0.18%
Dihydrokaempferol	85371.9844	0.08%	179953.297	0.49%
5-Hydroxy-3′,4′-dimethoxyflavanone	701131.375	0.69%	164051.016	0.45%
Epigallocatechin	47748.1445	0.05%	14032.4619	0.04%
8-Prenylnaringenin	122367.898	0.12%	26125.9199	0.07%
Hesperetin-7-O-neohesperidoside	461682.781	0.46%	24730.291	0.07%
Diosmin	338437.313	0.33%	13721.9141	0.04%
Myricetin-3-O-hexoside	15238.1211	0.02%	37693.0391	0.10%
Myricetin 3-glucoside	26058.6191	0.03%	9569.81836	0.03%
Apigenin-7-O-glucoside	427129.656	0.42%	17844.2559	0.05%
Chrysoeriol 7-O-glucoside	6,746,391	6.67%	1671090.88	4.58%
3’-Hydroxygenkwanin	120189.961	0.12%	183876.063	0.50%
Cyanidin-3-glucoside chloride	232028.109	0.23%	108885.445	0.30%
Isokurarinone	81933.75	0.08%	61272.168	0.17%
Kaempferide	676359.938	0.67%	151079.594	0.41%
Sakuranetin	81933.75	0.08%	23924.1035	0.07%
4′,7-Di-O-methylnaringenin	270447.438	0.27%	56605.875	0.16%
Tetramethylscutellarein	117476.461	0.12%	190626.797	0.52%
Epicatechin	67301.2344	0.07%	115823.18	0.32%
Narirutin	149367.391	0.15%	25765.3145	0.07%
3,5,6,7,8,3′,4′-Heptamethoxyflavone	61172.1406	0.06%	46100.7305	0.13%
Naringenin	487530.344	0.48%	23341.1543	0.06%
Licoflavone A	121610.266	0.12%	65017.9609	0.18%
5,7,4′-Trihydroxy-8-methylflavanone	39048.6836	0.04%	65017.9609	0.18%
Luteolin	833160.875	0.82%	35103.8711	0.10%
(-)Catechin	33823.5547	0.03%	37357.4688	0.10%
Hexamethylquercetagetin	66176.0313	0.07%	37914.5898	0.10%
Sinensetin	68733.3828	0.07%	161515.406	0.44%
Licoflavanone	307592.875	0.30%	48592.7188	0.13%
Isosinensetin	78448.1719	0.08%	2613621.5	7.16%
Isorhamnetin	46375.3711	0.05%	10876.8135	0.03%
3,7-Dihydroxy-3′,4′-dimethoxyflavone	92909.1719	0.09%	424557.094	1.16%
Trilobatin	40594.0781	0.04%	25810.8418	0.07%
Isopongaflavone	41022.7617	0.04%	10537176	28.88%
Halopemide	73232.5625	0.07%	41041.0977	0.11%
Liquiritigenin	1100135.13	1.09%	31535.0039	0.09%
Naringenin-7-O-glucoside	1074625.88	1.06%	1320291.75	3.62%
Phloretin-2′-O-glucoside	477843.938	0.47%	8,491,623	23.27%
Liquiritin	320917.906	0.32%	99693.4375	0.27%
Isoliquiritin	42475.7969	0.04%	97189.0938	0.27%
Pachyrrhizin	16957.1973	0.02%	55406.4375	0.15%
(-)-Epigallocatechin	47748.1445	0.05%	14032.4619	0.04%
Tiliroside	62905.9492	0.06%	59886.8516	0.16%
Formononetin	78,957,952	78.12%	1,860,036	5.10%
Isopsoralidin	42475.7969	0.04%	37885.2734	0.10%
Calycosin	604427.25	0.60%	2753168.25	7.54%
Glyasperin C	27202.1523	0.03%	298988.938	0.82%
Glabridin	13182.3125	0.01%	22023.3027	0.06%

^*^Relative content (%) = (Ai/ΣAi) * 100%, Ai, peak area of the shared compound; ΣAi, total peak area of all shared compounds.

### 3.3 Antioxidant activities

**Figure 2:**
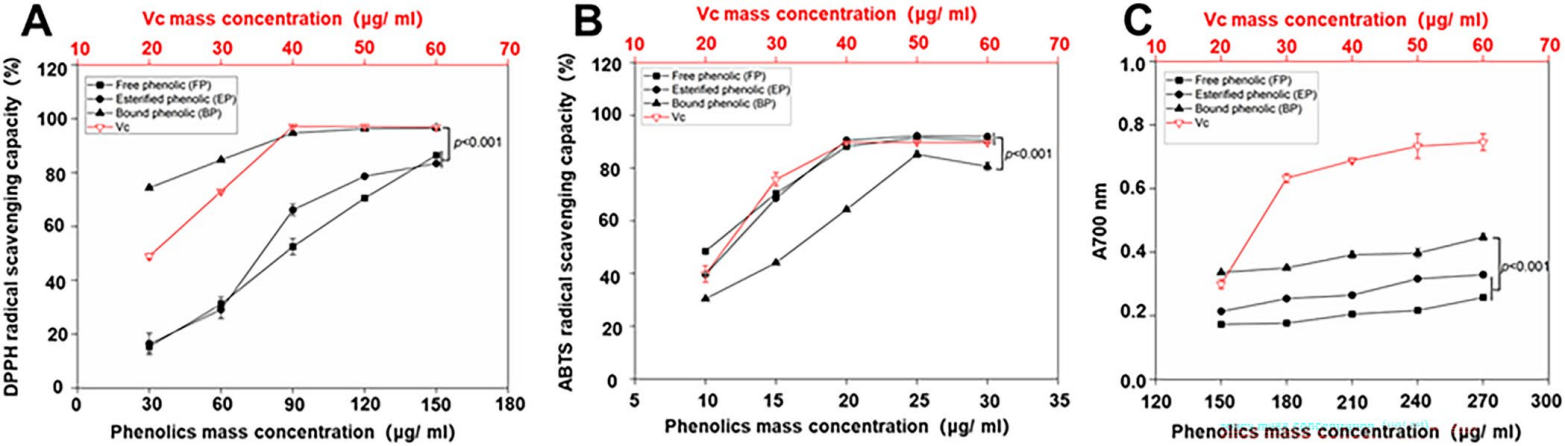
DPPH **(A)** and ABTS **(B)** radical-scavenging capacities and ferric ion reduction capacities **(C)** of different phenolic extracts. Data represent mean values for each sample ± deviations (*n* = 3). A two-way ANOVA test was performed, and statistically significant differences were compared.

In this assay, Vc was used as a positive control. The DPPH scavenging activities of the free, esterified, and bound phenolics and Vc are shown in Figure 2. Figure 2A shows that the FP, EP, BP, and Vc leaves exhibited potent radical-scavenging activities in a dose-dependent manner. The bound phenolics (IC50: 14.21 ± 2.38 μg/mL) had the best DPPH radical-scavenging activity, followed by esterified (IC50: 74.49 ± 5.93 μg/mL) and free phenolics (IC50: 81.22 ± 5.3 μg/mL). IC50 values are listed in Table 4. The results obtained in this study indicate a statistically significant difference (*p* < 0.001) in the DPPH radical-scavenging activities of BP and other phenolics. Furthermore, the DPPH radical-scavenging activity of Vc (IC50: 20.58 ± 0.97 μg/mL) was lower than BP.

**Table 4:** DPPH and ABTS radical-scavenging capacities of different phenolic extracts.

Free radicals	Samples			Positive control
	Free	Esterified	Bound	L-Ascorbic acid (Vc)
DPPH (IC50 in μg/mL)	81.22 ± 5.3	74.49 ± 5.93	14.21 ± 2.38	20.58 ± 0.97
ABTS (IC50 in μg/mL)	10.17 ± 0.32	11.51 ± 0.28	16.19 ± 1.1	22.11 ± 1.26

Data represent mean values for each sample ± deviations (*n* = 3).

As shown in Figure 2B, the ABTS radical-scavenging capacities of the samples and Vc were investigated. Among the three phenolics, BP (IC50:16.19 ± 1.1 μg/mL) exhibited slightly lower ABTS radical-scavenging capacity compared to FP (IC50:10.17 ± 0.32 μg/mL) and EP (IC50: 11.51 ± 0.28 μg/mL). Though there was a significant difference observed between BP and FP (*p* < 0.001), the results indicated that the three phenolics had stronger ABTS radical-scavenging capacity than the positive control (Vc; IC50: 22.11 ± 1.26 μg/mL), which proves that BP has a good ABTS scavenging ability.

FRAP is an efficient method for determining the antioxidant capacity (25). In the FRAP assay, Vc was used as a positive control. The FRAP activities of free, esterified, and bound phenolics and Vc were investigated (Figure 2). As shown in Figure 2C, the reducing power of the samples and the Vc exhibited a concentration-dependent relationship. BP exhibited the highest absorption, whereas FP had the lowest contribution among the three phenolics under consideration. Furthermore, the results demonstrated that BP exhibited significantly higher (*p* < 0.001) absorption than EP and FP did. However, none of the phenolic compounds exhibited higher performance than Vc in the FRAP assay.

### 3.4 Cytotoxicity and anti-oxidative stress activity

**Figure 3:**
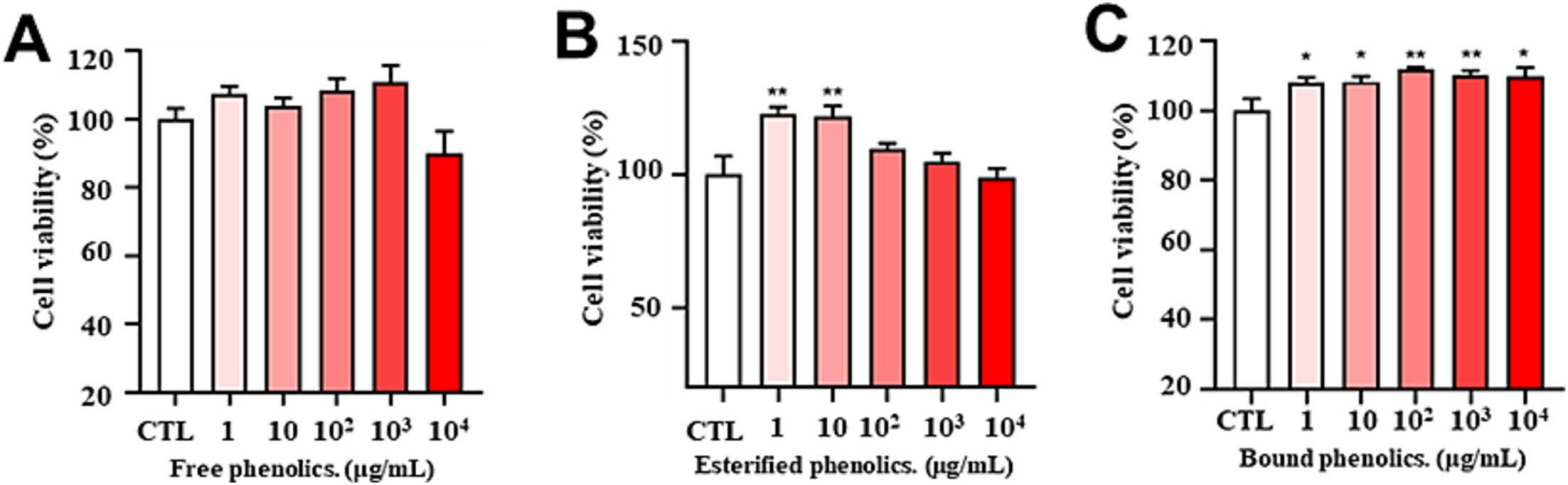
Cytotoxicity of different concentrations of free **(A)**, esterified **(B)**, and bound **(C)** phenolics. Cell viability was evaluated using the CCK-8 assay of PC12 cells treated for 24 h with phenolic extracts of AR at different concentrations (1, 10, 100, 1,000, and 10,000 μg/mL). The data are expressed as the percentage of viability of control cells. Data are shown as the mean ± SD (*n* = 3). One-way ANOVA was performed, followed by Tukey’s test. Statistically significant differences compared with the control group (cells without treatment) (^*^p < 0.05, ^**^p < 0.01, ^***^p < 0.001). CTL: group without H_₂_O_₂_ and BP treatment.

First, the cytotoxicity of free, esterified, and bound phenolic compounds was evaluated using the CCK-8 assay. Cell viability was calculated in relation to that of the control cells, which were considered to have 100% viability. As demonstrated in Figure 3A, FP showed low cytotoxicity even at a high concentration (10,000 μg/mL) for 24 h. Figure 3B further shows that EP was less cytotoxic at concentrations ranging from 1 to 10,000 μg/mL, while concurrently demonstrating the capacity to promote cell proliferation at concentrations as low as 1–10 μg/mL. Furthermore, Figure 3C demonstrates that BP was less cytotoxic and possessed the capacity to promote cell proliferation at concentrations ranging from 1 to 10,000 μg/mL.

**Figure 4:**
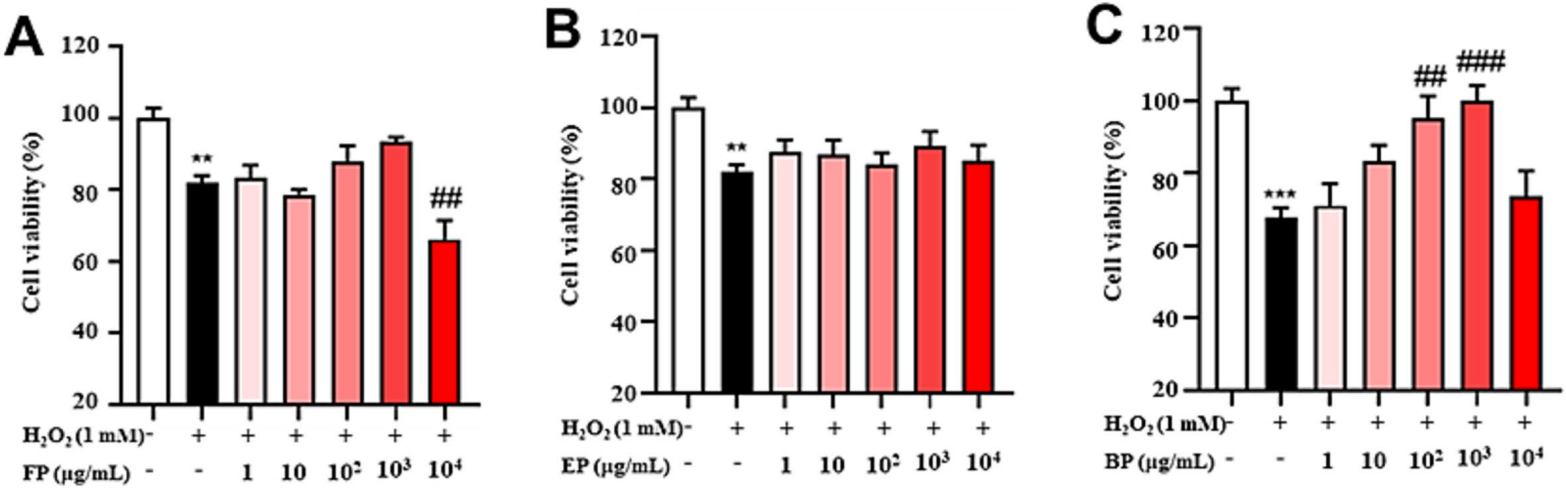
Phenolic extracts of astragalus residues prevent H_2_O_2_-induced cellular oxidative stress. **(A)** free phenolics, **(B)** esterified phenolics, **(C)** bound phenolics. Cell viability was evaluated by the CCK-8 assay of PC12 cells treated with H_2_O_2_ or phenolic extracts of astragalus residues at different concentrations (1, 10, 100, 1,000, and 10,000 μg/mL) for 24 h. The data are expressed as the percentage of viability of control cells. Data are shown as the mean ± SD (*n* = 3). One-way ANOVA was performed, followed by Tukey’s test. Statistically significant differences compared to the control group (untreated cells). ^*^Indicated significant difference (^*^p < 0.05, ^**^p < 0.01, ^***^p < 0.001) between control and H_2_O_2_ groups. ^#^Represented a significant difference between H_2_O_2_ and phenolic groups (^#^*p* < 0.05, ^##^*p* < 0.01, ^###^*p* < 0.001). CTL, group without H_₂_O_₂_ and BP treatment.

Subsequently, the antioxidant activity of the phenolic compounds was determined under conditions equivalent to those used in experiments conducted to evaluate cellular toxicity. As shown in Figure 4A, there was a significant decrease in cell viability in the H_2_O_2_-induced group in comparison with that in the control group (*p* < 0.01). No significant changes were observed in the FP-administered group within the 1–1,000 μg/mL concentration range. However, a significant reduction in cell viability was observed at a concentration of 10,000 μg/mL compared to that in the H_2_O_2_-induced group (*p* > 0.05). As shown in Figure 4B, the cell viability of the H_2_O_2_-induced group was significantly lower than the control group (*p* < 0.01). In the EP-administered group, no significant change was observed in the 1–10,000 μg/mL concentration range compared with the H_2_O_2_-induced group (*p* > 0.05). However, the results in Figure 4C indicate that cell viability was dramatically reduced in the H_2_O_2_-induced group compared with that in the control group (*p* < 0.001). Conversely, BP administration at concentrations ranging from 100 to 1,000 μg/mL resulted in a significant increase in cell viability compared to that in the H_2_O_2_-induced group (*p* < 0.01). The results indicated that BP could alleviate H_2_O_2_-induced oxidative stress in PC12 cells within a concentration range of 100–1,000 μg/mL.

### 3.5 Alleviation on H_2_O_2_-induced oxidative stress in PC12 cells

**Figure 5:**
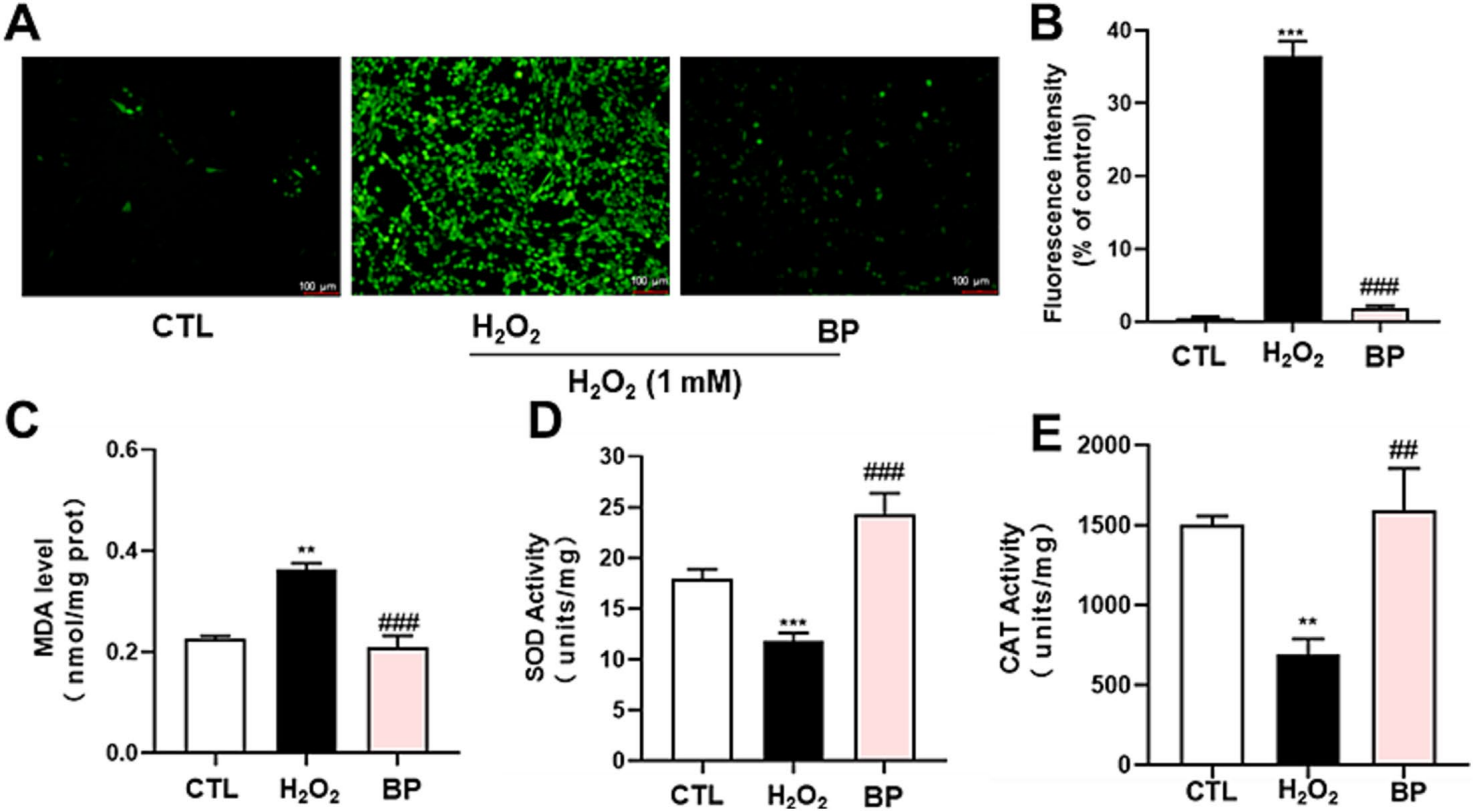
Phenolic extracts of astragalus residue reduce ROS **(A,B)** and MDA **(C)** produced by H_2_O_2_-induced oxidative stress by increasing SOD **(D)** and CAT **(E)** levels in PC12 neuronal cells. The data are expressed as the percentage of viability of control cells. Data are shown as the mean ± SD (*n* = 3). One-way ANOVA was performed, followed by Tukey’s test. Statistically significant differences compared to the control group (untreated cells). ^*^Indicated significant difference (^*^p < 0.05, ^**^p < 0.01, ^***^p < 0.001) between control and H_2_O_2_ groups. ^#^Represented a significant difference between H_2_O_2_ and phenolic groups (^#^*p* < 0.05, ^##^*p* < 0.01, ^###^*p* < 0.001). CTL, group without H_₂_O_₂_ and BP treatment.

Subsequently, the protective effects of BP against oxidative stress induced by H_2_O_2_ were investigated. These results indicated that substantial ROS levels were initially generated in PC12 cells upon exposure to H_2_O_2_. Subsequent treatment with BP (100 μg/mL) resulted in a marked decrease in the fluorescence intensity (Figure 5A). As shown in Figure 5B, the fluorescence intensity of the H_2_O_2_ group was over 35 times stronger than that of the CTL group (group without H_2_O_2_ and BP treatment), whereas the fluorescence intensity of the BP group was significantly lower than that of the H_2_O_2_ group.

As shown in Figure 5C, the amount of MDA was approximately 0.2 nmol/mg in PC12 cells and was observed to be increased significantly (*p* < 0.01) in the H_2_O_2_ group. Conversely, treating PC12 cells with BP led to a significant reduction in MDA levels (*p* < 0.001).

Our data also showed that the activities of several antioxidant enzymes (SOD and CAT) were higher in the BP group than in the H_2_O_2_ group (Figures 5D,E). Figure 5D shows that SOD activity was significantly decreased when induced by H_2_O_2_ compared to that in the CTL group, and the SOD activity in the BP group was significantly increased compared to that in the H_2_O_2_ group. Figure 5E shows that CAT activity showed the same trend as SOD after induction with H_2_O_2_. After BP treatment, CAT activity was greatly increased compared with that in the H_2_O_2_ group, suggesting that bound phenolics (100 μg/mL) had transcendent resistance to oxidative stress in PC12 cells.

### 3.6 Reduction of oxidative stress-related gene expression in PC12 cells

**Figure 6:**
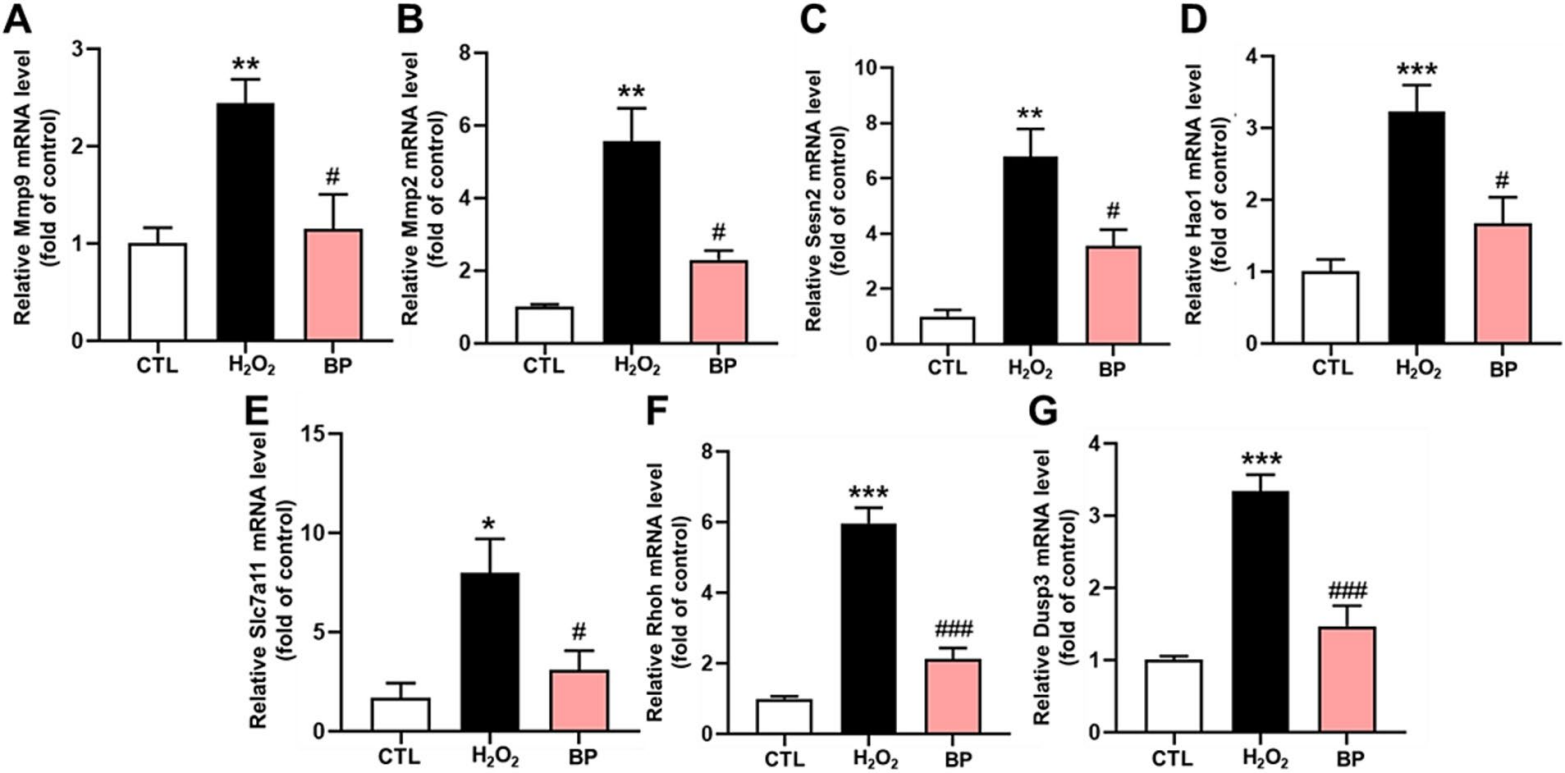
BP in AR reduced the expression of oxidative stress-related genes in PC12 cells. **(A–G)** represents Mmp9, Mmp2, Sesn2, Hao1, Slc7a11, Rhoh and Dusp3 genes respectively. Data are shown as the mean ± SD (*n* = 6). Tukey’s test was performed. Statistically significant differences compared to the control group (untreated cells). ^*^Indicated significant difference (^*^p < 0.05, ^**^p < 0.01, ^***^p < 0.001) between control and H_2_O_2_ groups; ^#^Represented a significant difference between H_2_O_2_ and BP groups (^#^*p* < 0.05, ^##^*p* < 0.01, ^###^*p* < 0.001). CTL, group without H_2_O_2_ and BP treatment.

The MMP9 mRNA level increased significantly after H_2_O_2_ stimulation (*p* < 0.01) and decreased significantly after BP treatment (*p* < 0.05) (Figure 6A). Also, the MMP2 mRNA level increased significantly following H_2_O_2_ stimulation (*p* < 0.01) and decreased significantly following BP treatment (*p* < 0.05) (Figure 6B). Figure 6C indicates that the Sesn2 mRNA level increased significantly after H_2_O_2_ stimulation (*p* < 0.01) and decreased significantly after BP treatment (*p* < 0.05). Also, Figure 6D indicated that the Hao1 mRNA level increased significantly after H_2_O_2_ stimulation (*p* < 0.001) and decreased significantly after BP treatment (*p* < 0.05). In line with this, the Slc7a11 mRNA level increased significantly following H_2_O_2_ stimulation (*p* < 0.05) and decreased significantly following BP treatment (*p* < 0.05) (Figure 6E). Figure 6F showed that the Rhoh mRNA level increased significantly after H_2_O_2_ stimulation (*p* < 0.001) and decreased significantly after BP treatment (*p* < 0.001). Figure 6G suggested that the Dusp3 mRNA level increased significantly after H_2_O_2_ stimulation (*p* < 0.001), and decreased significantly after BP treatment (*p* < 0.001), indicating that bound phenolics (100 μg/mL) exhibited a remarkable degree of resistance to oxidative stress in PC12 cells.

## 4 Discussion

This study focused on *Astragalus membranaceus* residue (AR), investigating and comparing the antioxidant efficacy of three phenolic fractions and identifying their bioactive components to achieve high-value utilization of this byproduct. The proposed strategy not only alleviates environmental pressures caused by herbal residue disposal but also reveals that its abundant insoluble-bound phenolics can serve as natural antioxidants for applications in the food preservation and feed additive sectors. This approach significantly enhances resource utilization efficiency by transforming agricultural waste into functional materials with industrial potential.

### 4.1 Effects of different extraction methods on the FP, EP, and BP contents

The phenolic yield is related to the concentration, type, polarity, extraction time, pH, temperature, and liquid-feed ratio of the solvent. The extraction solvent and chemical nature of the sample have the most important influence at the same time and temperature (35). The extraction of phenolic compounds using single solvents (36–39) vs. mixed solvents (diethyl ether-ethyl acetate) (23, 24, 40) shows different trends across various botanical matrices, indicating that neither method is absolutely superior. This variation is likely due to the inherent differences in total phenolic contents among plant species, with particularly notable differences in the amount of bound phenolics. To optimize the extraction of bound phenolics for subsequent *in vitro* antioxidant capacity tests, two extraction strategies were systematically employed. Therefore, in this study, by comparing the extraction effects of the two solvent systems on FP, EP, and BP in AR, it was found that the solubilization efficiency and total content of the three phenolics using mixed solvents (ethyl acetate and anhydrous ether) were significantly superior to those of a single solvent. This suggests that the synergistic effect of mixed solvents can more effectively disrupt the phenolic-macromolecular composite structure, thereby releasing more active ingredients.

### 4.2 Analysis of the composition and relative content of phenolic compounds

Multiple novel phenolic compounds were identified from green tea (41), ginkgo leaves (42), olive oil (43), and medicinal plants (44) using UHPLC-Q-TOF MS because it enables the rapid, high-resolution separation and accurate structural identification of phenolic compounds via sub-2 μm column-driven efficiency (theoretical plates > 100,000/m), high-pressure operation (1,000 bar) reducing analysis time by 60–80%, and high-precision mass spectrometry (Δm < 1.5 mDa) for molecular formula inference and MS/MS-based isomer differentiation (41, 45, 46). Therefore, UHPLC Q-TOF-MS was used to identify the phenolic compounds in the three fractions, which showed that the types of phenolic compounds in EP are fewer than those in FP and BP, which also suggests the reason why EP has weaker antioxidant capacity. The three fractions had very few phenolic compounds in common is mainly due to the different chemical binding states of their phenolic hydroxyl groups. The phenolic hydroxyl group (–OH) of FP exists freely and is not bound to other functional groups; The phenolic hydroxyl group of EP is connected to fatty acids, organic acids, etc. through ester bonds (–COO–); The phenolic hydroxyl groups of BP bind to macromolecules such as polysaccharides and proteins through glycosidic, ester, or covalent bonds. The total number of phenolic compounds contained in BP (115) is slightly less than that in FP (137), but its subsequent *in vitro* antioxidant capacity is superior to that of FP. Therefore, the specific compounds were further analyzed. Phenolic compounds can be classified into three types: phenolic acids, flavonoids, and isoflavonoids. The UHPLC Q-TOF-MS results indicate that flavonoids in BP, including catechins (47), camptothecin (48), hesperidin (49), isosinensein (50), and calycosin (51), have been shown to have strong antioxidant capacity. It is worth mentioning that the relative content of catechin, isosinensein, and calycosin in BP is higher than that in FP, which may also make it have stronger antioxidant capacity. Moreover, the stronger *in vitro* antioxidant capacity of BP may also be further enhanced due to the synergistic effect between phenolic compounds, as the synergistic effects of catechins and other antioxidants have been demonstrated (52, 53).

### 4.3 Evaluation of antioxidant activity *in vitro*

*In vitro* antioxidant assays demonstrated that BP exhibited significantly superior DPPH radical-scavenging capacity than FP, EP, and the positive control (Vc). BP also exhibited an enhanced ferric ion-reducing power relative to the other fractions. These findings align with those of previous studies on blueberry seed meal (23) and chickpeas (24) and confirm the optimal antioxidant efficacy of BP. Contrasting observations have been reported for walnut kernels, where free phenolics demonstrated the strongest *in vitro* antioxidant activity (54), suggesting that species-specific variations may influence bioactivity distribution among phenolic fractions. Cytotoxicity evaluation using PC12 neuronal cells revealed no significant toxicity of the three phenolic fractions within a concentration range of 0–10,000 μg/mL. At concentrations of 100–1,000 μg/mL, BP effectively alleviated H_2_O_2_-induced oxidative stress, as evidenced by significantly reduced intracellular ROS and MDA levels, accompanied by markedly elevated SOD and CAT enzyme activities. BP reduced the fluorescence intensity of ROS in PC12 induced by H_2_O_2,_ which increased by approximately 35 times compared to the control group. Consistent with this, phenolic acids and flavonoids-rich *Glechoma hederacea* L.(Lamiaceae) water extract reduced the counts of ROS induced by H_2_O_2,_ which increased by approximately 1.3 times compared to the control group (55). The results also showed that BP significantly decreased the amount of MDA, which was approximately half of the H_2_O_2_ group. Moreover, BP significantly increased the activity of SOD and CAT which was approximately twice of the H_2_O_2_ group. This is consistent with the results that phenolics in Rhododendron anthopogonoides (63) significantly increased the activities of SOD and CAT in PC12 to twice that in H_2_O_2_ group. At the same time, intracellular ROS accumulation promotes transcription of the Mmp9 (56), Mmp2 (57), Sesn2 (58), Hao1 (59), Slc7a11 (60), Rhoh (61) and Dusp3 (62), and BP in AR could down-regulate the expression of these genes, indicating that BP has a certain antioxidant capacity.

### 4.4 Limitations and future research directions

The study recommends further absolute quantitative research on specific phenolic compounds in FP, EP, and BP to accurately elucidate the compounds that exert antioxidant effects. Furthermore, to validate the translatability of the *in vitro* antioxidant findings reported in this study to physiological conditions, future research should focus on conducting *in vivo* studies with BP in AR. Investigating the potential for antioxidant activities observed in cell-free systems to be replicated in living organisms is essential. Such research is instrumental in bridging the gap between *in vitro* findings and *in vivo* efficacy, thereby providing a theoretical foundation for the exploration of new antioxidants.

## 5 Conclusion

Polyphenols are specific nutrients that have been proven to have a positive effect on a range of brain disorders and are thus regarded as promising therapeutics. High-value components recovered from AR, such as natural antioxidants, can be economically and efficiently used in nutraceuticals, functional foods, and dietary supplements. AR has unique advantages owing to its abundant insoluble-bound phenolic fraction, which combines eco-friendly attributes with economic valorization potential. Systematic exploitation of this resource could substantially enhance comprehensive resource utilization efficiency while providing both theoretical foundations and technological frameworks for naturally occurring nutritional supplements.

## Data Availability

The original contributions presented in the study are included in the article/Supplementary material, further inquiries can be directed to the corresponding authors.
