# Supplementary material for: Antioxidant action and potential neuroprotection of polyphenolics extracted from Astragalus membranaceus residue
Source: Front Nutr. 2025 Jul 23;12:1621848. doi: 10.3389/fnut.2025.1621848 (PMC12326745; doi:10.3389/fnut.2025.1621848)
Supplement: Supplementary file 1 [file Data_Sheet_1.pdf]

Supporting Information for

Original article

Antioxidant action and potential neuroprotection of polyphenolics extracted from *Astragalus membranaceus* residue

Lu Li [1,2,†], Qiaona Wang [3,†], Ying Cao [1,2], Jianmei Li [1], Yulong Wu [2], Chun Hua [2], Feng Zhou [2], Shengjie Li [2,*], Su Liu [1,*]

1 School of Food Science and Pharmaceutical Engineering, Nanjing Normal University, Nanjing 210023, People's Republic of China

2 School of Food Science, Nanjing Xiaozhuang University, Nanjing 211171, People's Republic of China

3 School of Ecology and Applied Meteorology, Nanjing University of Information Science & Technology, Nanjing 210044, People's Republic of China

* Authors to whom correspondence should be addressed.

† These authors contributed equally to this work.

This file includes:

1. Supporting tables (Table S1)

2. Supporting figures (Figure S1)

3. Supporting figures (Figure S2)

1. Supporting tables (Table S1)

Table S1. Phenolic composition of different extracts in astragalus residue using UHPLC Q-TOF-MS.

	Compounds	FP	EP	BP
Phenolic acid	4-Aminobenzoic acid	1	0	0
	2,5-Dihydroxybenzoate	1	0	0
	3-Amino-5-hydroxybenzoic acid	1	0	0
	Ethyl syringate	1	0	0
	Butamben	1	0	0
	N-acetyl mesalazine	1	0	0
	Mebeverine	1	0	0
	Di(2-methoxyethyl) phthalate	1	0	0
	Bergenin	1	0	0
	3,4-Dihydroxybenzoic acid	1	0	0
	6-Benzoylheteratisine	1	0	0
	Dihexyl phthalate	1	0	0
	3-Amino-4-hydroxybenzoic acid	1	0	0
	Hippuric acid	1	0	0
	Octinoxate	1	0	0
	4-Ethylbenzoic acid	1	0	0
	Salicylic acid	1	0	0
	2,6-Dihydroxybenzoic acid	1	0	0
	3,4-Dihydroxybenzoate	1	0	0
	trans-Cinnamic acid	1	0	0
	5-Methoxysalicylic acid	1	0	0
	2-Hydroxyphenylacetic acid	1	0	0
	p-Anisic acid	1	0	0
	3-Aminosalicylic acid	1	0	0
	2-Amino-3-methoxybenzoic acid	1	0	0
	Ginnalin B	1	0	0
	3-Methylorsellinic acid	1	0	0
	Olivetolcarboxylic acid	1	0	0
	Rhizonic acid	1	0	0
	Veratric acid	1	0	0
	Ctrinin hydrate	1	0	0
	3-Hydroxybenzoic acid	1	0	0
	Ethyl vanillate	1	0	0
	1,2-Bis(p-(2-hydroxyethoxycarbonyl)benzoyloxy)ethane	1	1	0
	4-Hydroxy-benzoate	1	1	0
	Benzoic acid	1	1	0
	Ozagrel	1	0	1

	Compounds	FP	EP	BP
**Phenolic acid**	Methyl gallate	1	0	1
	Syringic acid	1	0	1
	2-Methoxy-4-pentadecylbenzoic acid	1	0	1
	Benzamide	1	0	1
	Vanillic acid	1	0	1
	Halopemide	1	0	1
	Benzyl-butyl-phthalate	0	1	0
	Diethylene glycol dibenzoate	0	1	0
	Taprostene	0	1	1
	Bis(2-ethylhexyl)phthalate	0	1	1
	Di-n-butyl phthalate	0	1	1
	Benzyl butyl phthalate	0	1	1
	2,3-Dihydroxybenzoate	0	0	1
**Flavonoid**	Luteolin-4'-O-glucoside	1	0	0
	Apigenin	1	0	0
	Isorhamnetin-3-O-galactoside-6"-rhamnoside	1	0	0
	Ginkgetin	1	0	0
	Dihydrohesperetin-7-O-neohesperidoside	1	0	0
	Isorhamnetin-3-O-glucoside	1	0	0
	4',5,7-Trihydroxy-3,6-dimethoxyflavone	1	0	0
	7-Hydroxy-6-methoxydihydroflavonol	1	0	0
	3-Hydroxy-3',4'-dimethoxyflavone	1	0	0
	4',7-Dimethoxy-3-hydroxyflavone	1	0	0
	Silydianin	1	0	0
	Acaciin	1	0	0
	Glucoluteolin	1	0	0
	Kaempferol	1	0	0
	Pelargonidin 3-O-glucoside	1	0	0
	Thermopsoside, crotonoyl	1	0	0
	5,7-Dimethoxyflavanone	1	0	0
	4'-Methoxyflavone	1	0	0
	Wogonoside	1	0	0
	6-Methylflavonol	1	0	0
	Skullcapflavone II	1	0	0
	Kumatakenin	1	0	0
	Dihydroquercetin	1	0	0
	6-Prenylnaringenin	1	0	0
	Okanin-4'-O-glucoside	1	0	0
	Isopongaflavone	1	1	1
	(-)-Epigallocatechin	1	1	1

	Compounds	FP	EP	BP
Flavonoid	Tiliroside	1	1	1
	Liquiritigenin	1	0	1
	Naringenin-7-O-glucoside	1	0	1
	Phloretin-2'-O-glucoside	1	0	1
	Liquiritin	1	0	1
	Isoliquiritin	1	0	1
	Isokaempferide	1	0	1
	Astragalin	1	0	1
	Eriodictyol-7-O-glucoside	1	0	1
	7,3',4'-Trihydroxyflavone	1	0	1
	Apigenin-6-C-glucoside-7-O-glucoside	1	0	1
	Apigetrin	1	0	1
	2',6'-Dihydroxy-4-methoxychalcone-4'-O-neohesperid	1	0	1
	Luteolin 3',4'-dimethyl ether	1	0	1
	Flavanomarein	1	0	1
	Dihydrokaempferol	1	0	1
	5-Hydroxy-3',4'-dimethoxyflavanone	1	0	1
	Epigallocatechin	1	0	1
	8-Prenylnaringenin	1	0	1
	Hesperetin-7-O-neohesperidoside	1	0	1
	Diosmin	1	0	1
	Myricetin	1	0	1
	Apigenin-7-O-glucoside	1	0	1
	Chrysoeriol 7-O-glucoside	1	0	1
	3'-Hydroxygenkwanin	1	0	1
	Cyanidin-3-glucoside chloride	1	0	1
	Isokurarinone	1	0	1
	Kaempferide	1	0	1
	Sakuranetin	1	0	1
	4',7-Di-O-methylnaringenin	1	0	1
	4'-Hydroxy-5,7-dimethoxyflavanone	1	0	1
	Tetramethylscutellarein	1	0	1
	Epicatechin	1	0	1
	Narirutin	1	0	1
	3,5,6,7,8,3',4'-Heptamethoxyflavone	1	0	1
	Naringenin	1	0	1
	Licoflavone A	1	0	1
	5,7,4'-Trihydroxy-8-methylflavanone	1	0	1
	Luteolin	1	0	1
	(-)Catechin	1	0	1

	Compounds	FP	EP	BP
	Hexamethylquercetagetin	1	0	1
	Sinensetin	1	0	1
	licoflavanone	1	0	1
	Isosinensetin	1	0	1
	Isorhamnetin	1	0	1
	3,7-Dihydroxy-3',4'-dimethoxyflavone	1	0	1
	Trilobatin	1	0	1
	Jaceosidin	1	0	0
	Icaritin	0	1	0
	5,6-Benzoflavone	0	1	0
	8,8-Dimethyl-2-phenylpyrano[2,3-f]chromen-4-one	0	1	1
	Anhydroicaritin	0	1	1
	Kaempferol-3-O-glucoside-6''-p-coumaroyl	0	1	1
	Hyperoside	0	0	1
	Catechin(+)	0	0	1
	Maritimein	0	0	1
	Apigenin-7-O-neohesperidoside	0	0	1
	Phlorizin	0	0	1
	Kaempferol-7-O-beta-D-glucopyranoside	0	0	1
	Cosmosiin Apigenin 7-O-glucoside	0	0	1
**Flavonoid**	Chrysin dimethyl ether	0	0	1
	Pinocembrin	0	0	1
	Artemitin	0	0	1
	Bullatine G	0	0	1
	Naringin dihydrochalcone	0	0	1
	(2R,3R)-3,7,4'-Trihydroxy-5-methoxy-8-prenylflavanone	0	0	1
	Naringenin-7-O-beta-D-glucoside	0	0	1
	Wogonin	0	0	1
	Isoquercitrin	0	0	1
	2''-Rhamnosylvitexin	0	0	1
	Galangin	0	0	1
	Alpinetin	0	0	1
	Chrysin 7-O-beta-gentiobioside	0	0	1
	3,3'-Dihydroxyflavone	0	0	1
	3',4',7-Trihydroxyflavanone	0	0	1
	Cirsimaritin	0	0	1
	Fisetinidol	0	0	1
	Kuwanon C	0	0	1
	Corymbosin	0	0	1
	Hesperetin	0	0	1

	Compounds	FP	EP	BP
Flavonoid	Diosmetin	0	0	1
	Acacetin	0	0	1
	Velutin	0	0	1
	5,6-Dihydroxy-3',4'-dimethoxyflavanone	0	0	1
	Isosakuranin	0	0	1
	Cirsimarin	0	0	1
	Isosakuranetin	0	0	1
	5-Hydroxy-2-(4-hydroxyphenyl)-7-methoxy-4H-chromen-4-one	0	0	1
	Eupafolin	0	0	1
	Hispiduloside	0	0	1
	Gardenin B	0	0	1
	Nobiletin	0	0	1
	Poncirin	0	0	1
	Malvidin	0	0	1
Isoflavone	Koparin	1	0	0
	Coumestrol	1	0	0
	7-Methoxy-2-methyl-3-phenyl-4H-chromen-4-one	1	0	0
	Amorphigenin	1	0	0
	Daidzein-8-C-glucoside	1	0	0
	5,7-dihydroxy-3-phenyl-4H-chromen-4-one	1	0	0
	Flemiphilippinin A	1	0	0
	(-)-Medicarpin	1	0	0
	7,8-dihydroxy-3-(4-hydroxyphenyl)-4H-chromen-4-one	1	0	0
	Irigenin, 7-benzyl ether	1	0	0
	Corylin	1	0	0
	Formononetin	1	1	1
	Isopsoralidin	1	1	1
	Calycosin	1	1	1
	Glyasperin C	1	1	1
	Glabridin	1	1	1
	Pachyrrhizin	1	1	0
	Coumestrol dimethyl ether	1	0	0
	4,5,7-Trihydroxyisoflavone	0	0	1
	Puerarin	0	0	1
	Irisflorentin	0	0	1
	Glabrone	0	0	1

*1：this compound was contained   0: this compound was not contained

2. Supporting figures (Figure S1)

A

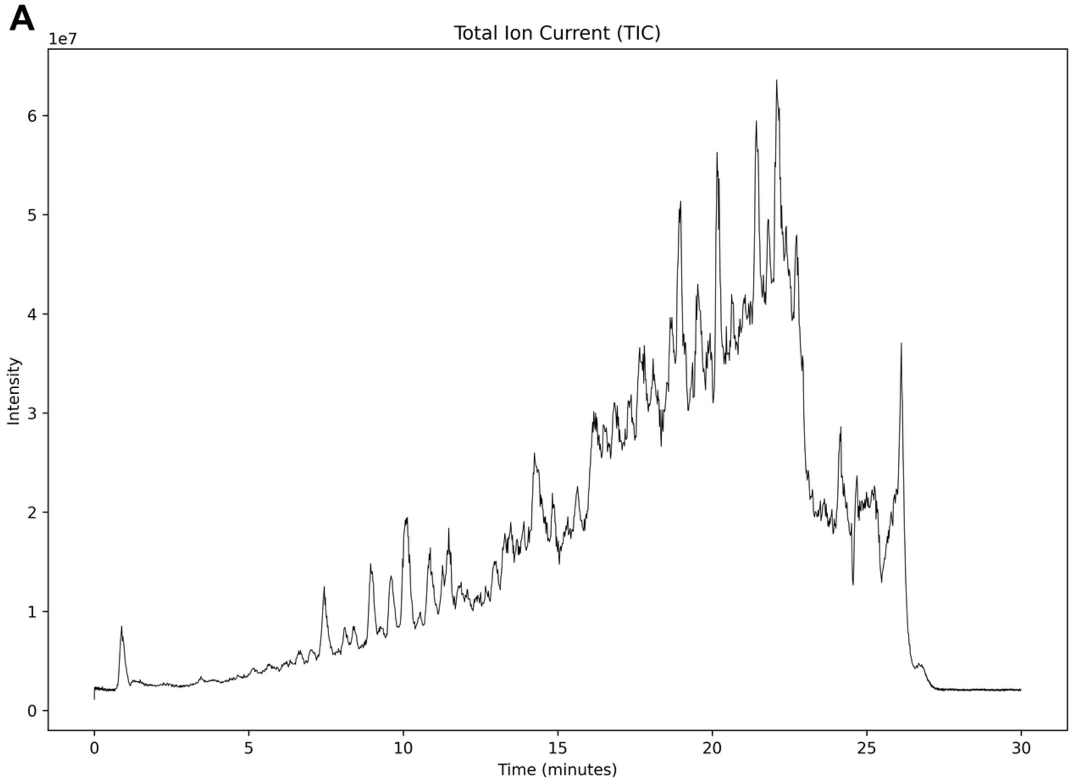

B

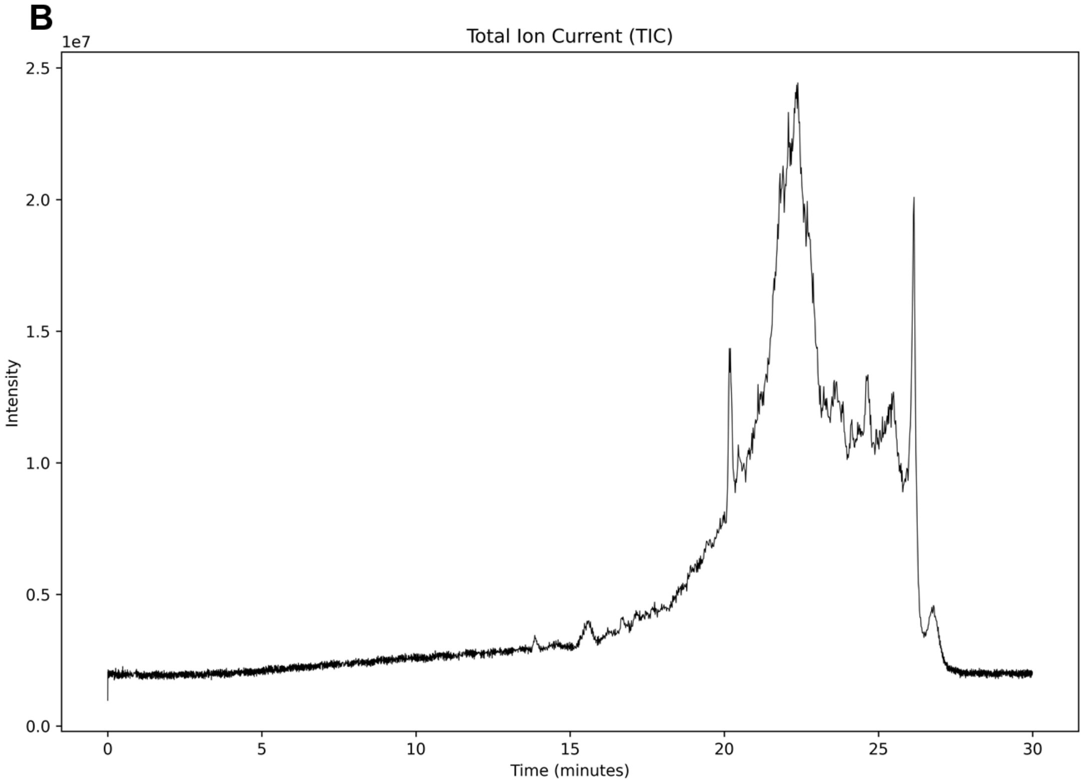

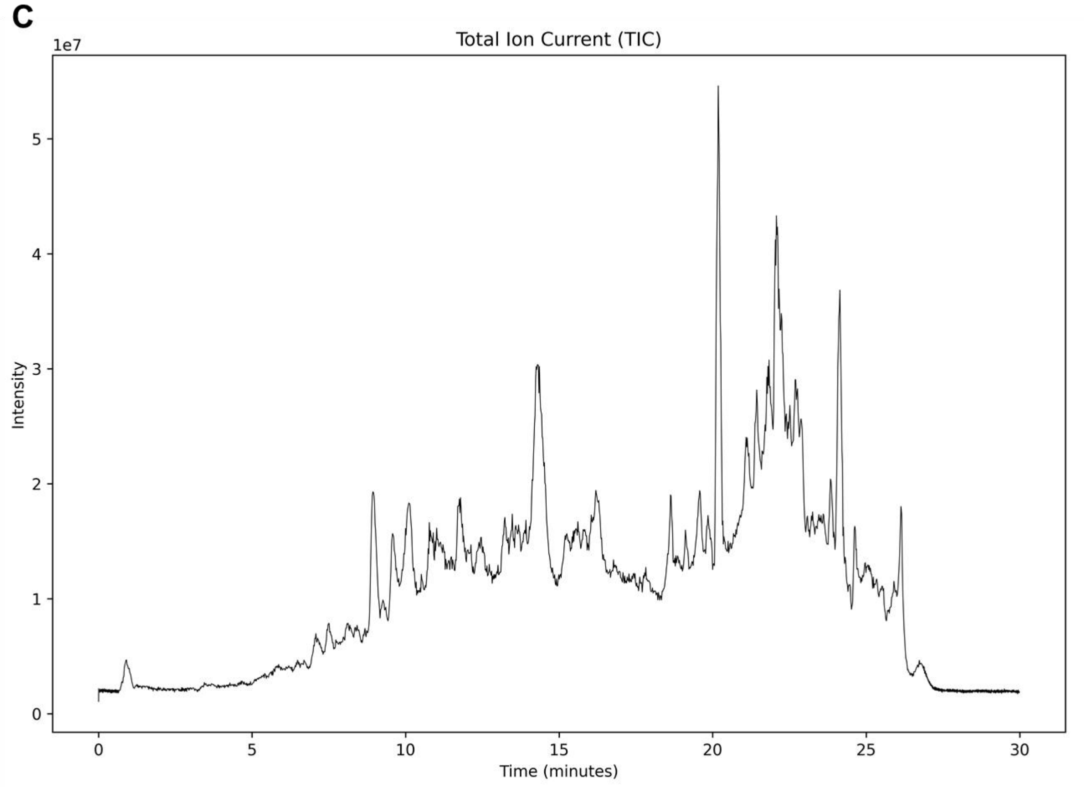

Figure S1. Total ion current (TIC) diagram in positive mode of the A- free, B- esterified and C- bound phenolics in *A membranaceus* residue.

3. Supporting figures (Figure S2)

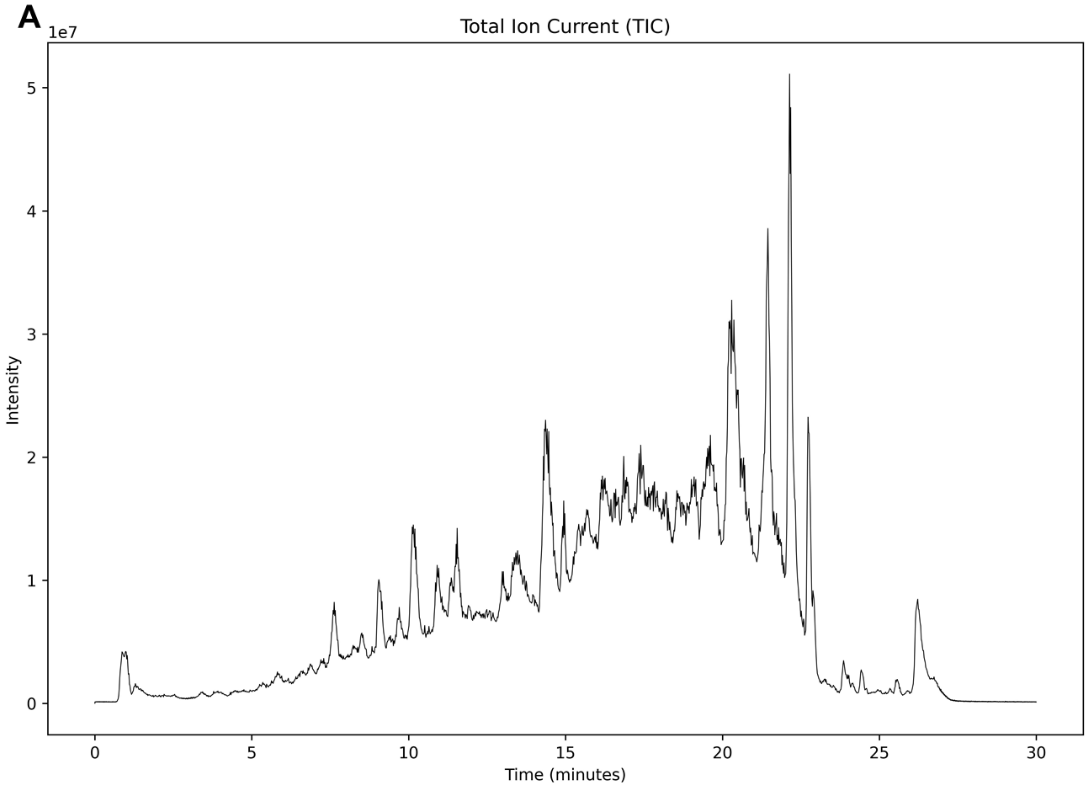

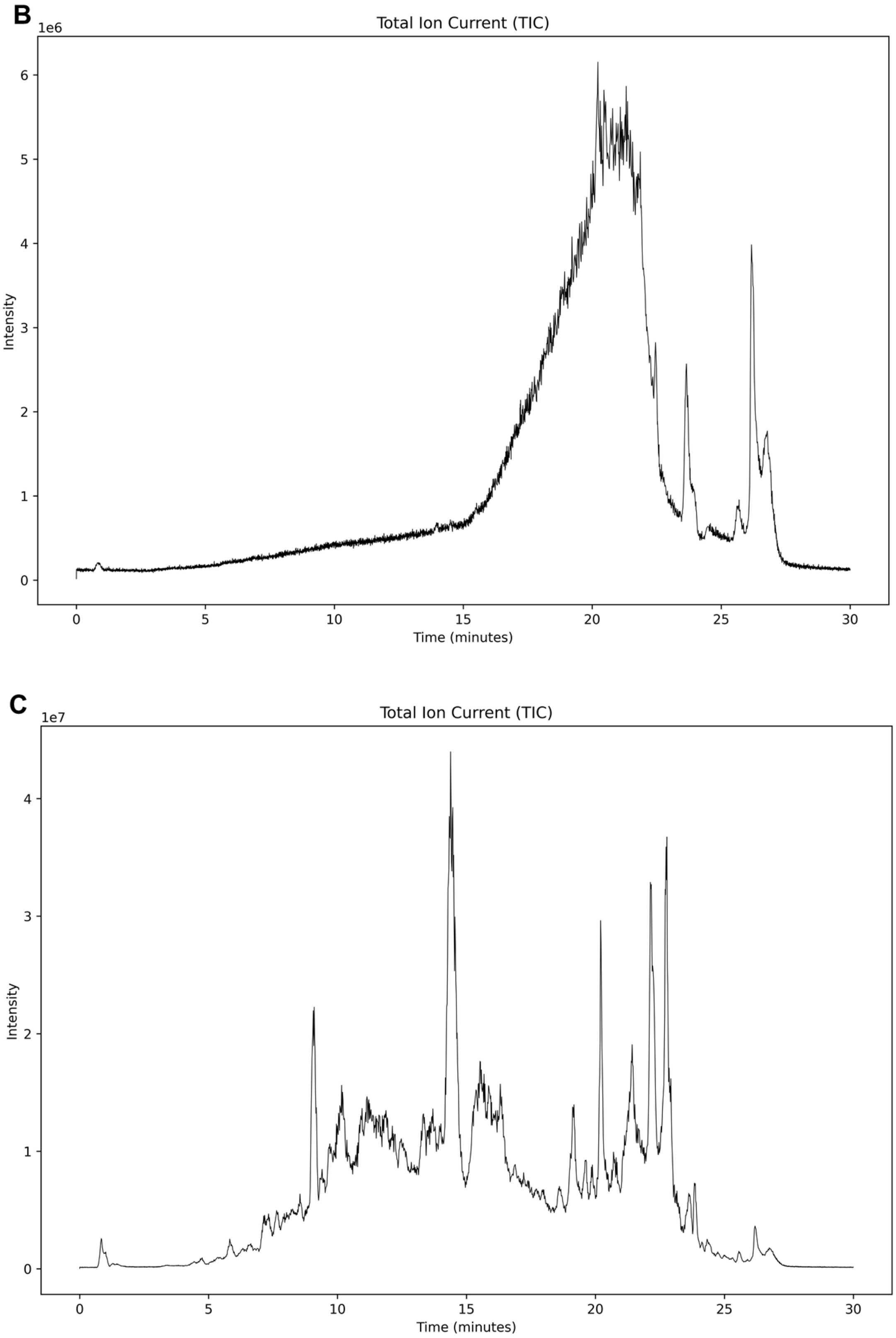

Figure S2. Total ion current (TIC) diagram in negative mode of the A- free, B- esterified and C- bound phenolics in *A membranaceus* residue.
